# Enzymatic degradation of polylactic acid (PLA)

**DOI:** 10.1007/s00253-024-13212-4

**Published:** 2024-07-10

**Authors:** Adi Shalem, Omer Yehezkeli, Ayelet Fishman

**Affiliations:** Department of Biotechnology and Food Engineering, Technion-Israel Institute of Technology, 3200003 Haifa, Israel

**Keywords:** Polylactic acid, Depolymerization, Biodegradation, Hydrolases, Upcycling

## Abstract

**Abstract:**

Environmental concerns arising from the increasing use of polluting plastics highlight polylactic acid (PLA) as a promising eco-friendly alternative. PLA is a biodegradable polyester that can be produced through the fermentation of renewable resources. Together with its excellent properties, suitable for a wide range of applications, the use of PLA has increased significantly over the years and is expected to further grow. However, insufficient degradability under natural conditions emphasizes the need for the exploration of biodegradation mechanisms, intending to develop more efficient techniques for waste disposal and recycling or upcycling. Biodegradation occurs through the secretion of depolymerizing enzymes, mainly proteases, lipases, cutinases, and esterases, by various microorganisms. This review focuses on the enzymatic degradation of PLA and presents different enzymes that were isolated and purified from natural PLA-degrading microorganisms, or recombinantly expressed. The review depicts the main characteristics of the enzymes, including recent advances and analytical methods used to evaluate enantiopurity and depolymerizing activity. While complete degradation of solid PLA particles is still difficult to achieve, future research and improvement of enzyme properties may provide an avenue for the development of advanced procedures for PLA degradation and upcycling, utilizing its building blocks for further applications as envisaged by circular economy principles.

**Key points:**

• *Enzymes can be promisingly utilized for PLA upcycling.*

• *Natural and recombinant PLA depolymerases and methods for activity evaluation are summarized.*

• *Approaches to improve enzymatic degradation of PLA are discussed.*

**Graphical Abstract:**

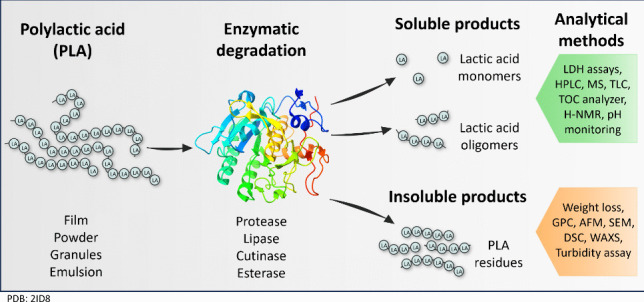

## Introduction

Over the years, researchers have extensively studied eco-friendly alternatives to traditional petroleum-based plastics. Biodegradable polymers such as polyhydroxyalkanoates (PHA), polybutylene succinate (PBS), polycaprolactone (PCL), and polylactic acid (PLA) are among them, with PLA being the most commonly used biodegradable polymer (Qi et al. 2017; Taib et al. 2023). In 2023, nearly 675 thousand tons of PLA were produced annually, accounting for around 31% of the total global bioplastic production capacity. With an expected threefold increase in global bioplastic production, PLA production is predicted to grow to approximately 3.2 million tons a year by 2028, representing about 43.6% of the total bioplastic production (European Bioplastics 2023). Compared to other polymers, PLA has exceptional properties such as durability, high mechanical strength, biocompatibility, transparency, and good processability. These qualities have made it a popular material for a wide range of applications across various industries, including being the primary material for 3D printing (Chen et al. 2016; Joseph et al. 2023). PLA is classified as Generally Recognized As Safe (GRAS) by the United States Food and Drug Administration (FDA), making it a preferred material in the food packaging industry (Swetha et al. 2023). In the textile industry, PLA’s characteristics such as good dyeability, low flammability, and water resistance make it a suitable substitute for traditional fabrics. In the biomedical industry, PLA is widely used in bone repair, sutures, drug delivery, and tissue engineering as a degradable scaffold (Chen et al. 2016; Singhvi et al. 2019). Additionally, in agriculture, it is utilized for mulch films and packaging since it is compostable and does not harm the soil (Zaaba and Jaafar 2020).

Lactic acid is the monomer comprising PLA. It can be produced by either chemical synthesis or by fermentation of renewable resources (corn, cassava, potato, sugar cane, etc.). Fermentation is the preferred process and is mostly used for lactic acid production, mainly by *Lactobacillus* bacteria, which exclusively produces lactic acid (Zaaba and Jaafar 2020). Compared to the production of petroleum-based plastics, it requires much less energy and emits fewer polluting gases (Qi et al. 2018; Teixeira et al. 2021). The lactic acid monomers are further polymerized into PLA by chemical methods, mainly direct polycondensation and ring opening polymerization (ROP). Direct polycondensation is accompanied by the elimination of water or alcohol byproducts and usually forms low molecular weight PLA of below 50,000 g/mol. ROP is performed through the formation of lactide, a cyclic dimer of lactic acid, and mostly forms higher molecular weight PLA. While organometallic catalysts are mostly used for ROP, enzymes such as lipase, have been reported as effective and eco-friendly biocatalysts for polymerization (Hu et al. 2016; Yadav et al. 2021). Eventually, PLA can be assimilated by microorganisms into biomass or carbon dioxide and water after use (Xu et al. 2022a).

**Fig. 1:**
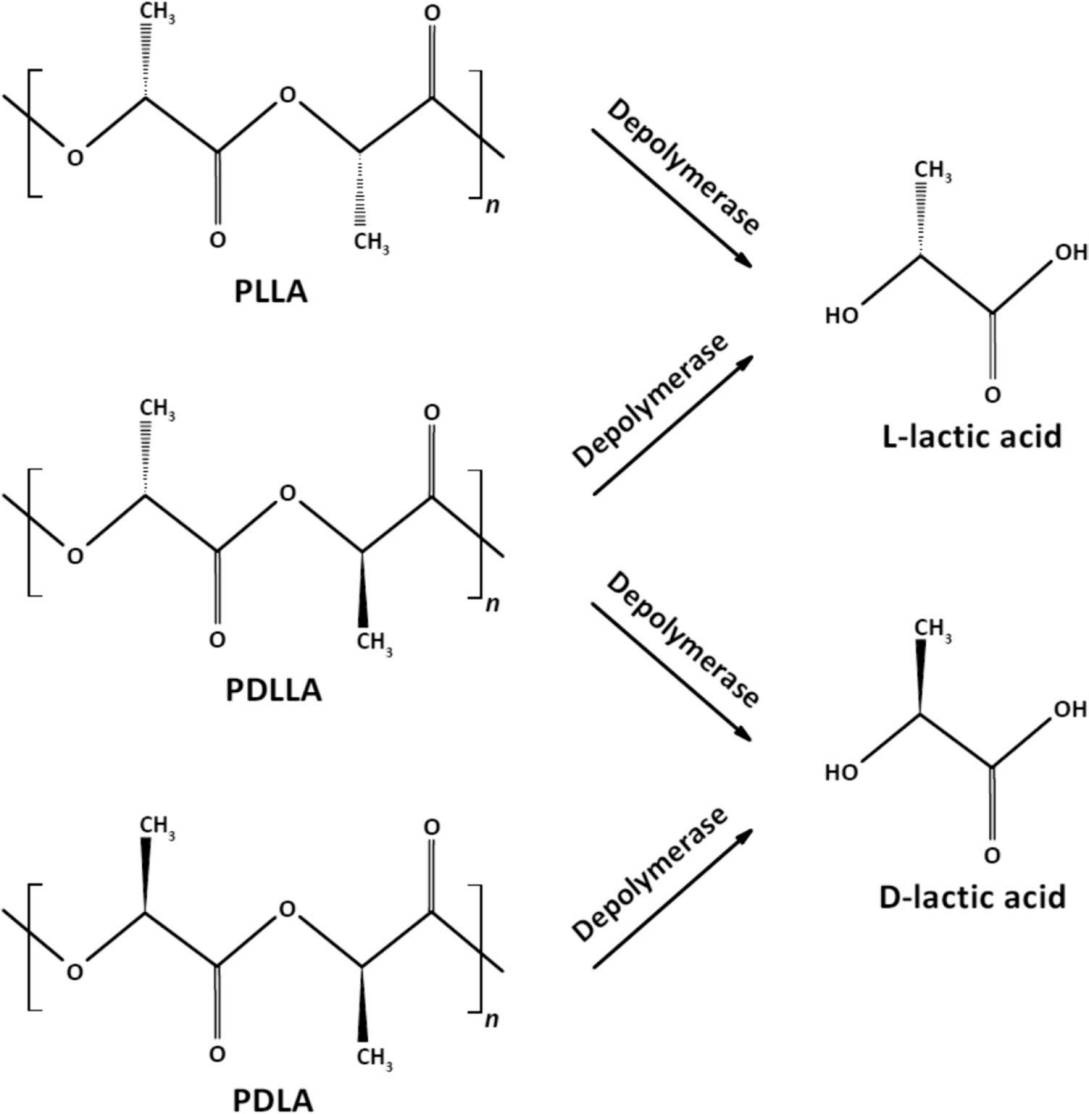
Enzymatic degradation of PLA stereochemical forms. Depolymerases include proteases, lipases, cutinases, and esterases

Depending on the chiral structure of the lactic acid isomers (L or D), PLA can exist in three stereochemical forms: poly-l-lactic acid (PLLA), poly-d-lactic acid (PDLA), or poly-d,l-lactic acid (PDLLA). The enantiomer composition affects the polymer crystallinity and thermal properties. Enantiomerically pure PLA (PLLA or PDLA) is usually semi-crystalline, while PDLLA is more amorphous (Taib et al. 2023). Generally, a racemic mixture of lactic acid monomers is produced when chemical synthesis is used, while enantiomerically pure lactic acid (l or d) is produced by microbial fermentation, depending on the selected strain (Singhvi et al. 2019). Commercial PLA usually consists of mostly L stereoisomers (Taib et al. 2023). To reduce crystallinity, enhance thermostability and improve mechanical properties, different percentages of d-lactic acid monomers are added to form stereocomplexed PLA (Luo et al. 2020; Huang et al. 2021; Kumar et al. 2022). Accordingly, PLA can be degraded into the corresponding monomers (Fig. 1). Another approach to improve PLA properties is reinforcing with other materials such as cellulose or PCL, to produce PLA-based composites (Li et al. 2023).

The extensive use of PLA raises the issue of waste disposal to minimize environmental impacts, which is expected to become more relevant and of concern with the projected increase in use over the next years. While mechanical and chemical recycling requires high energy consumption and waste cleaning, biodegradation-based processes can be more favorable. However, composting requires several months for complete degradation and was found to cause environmental impacts (Cosate de Andrade et al. 2016). While microbial degradation ends with the assimilation of valuable lactic acid, enzymatic degradation, using molecular, biocatalytic, and protein engineering techniques, offers a different approach. The use of enzymes can facilitate the efficient degradation and upcycling of PLA building blocks for further processes and applications. Moreover, a mild enzymatic hydrolysis of PLA can be used to increase hydrophilicity and expose functional reactive groups on its surface for different applications such as drug delivery (Ribitsch et al. 2012; Pellis et al. 2017). Yet, such use of enzymes still requires improvements and optimization to adapt to industrial processes and attain full degradation of large amounts of waste.

Zaaba and Jaafar (2020) and Teixeira et al. (2021) have reviewed various mechanisms of PLA degradation, which include chemical hydrolysis, photodegradation, microbial degradation, and enzymatic degradation. They have also examined PLA degradation under different conditions, such as composting and aquatic environments. Additionally, PLA biodegradation mechanisms have been reviewed by Qi et al. (2017) and Xu et al. (2022a), in which both microbial and enzymatic degradation were discussed. Qi et al. highlighted the establishment of simulated microbial degradation systems. Xu et al. detailed factors affecting biodegradation and focused on PLA upcycling using physical or mechanical recycling and biorecovery of secondary degradation products by microorganisms. Others also reviewed the use of enzymes for the synthesis and hydrolysis of various polyesters, including PLA (Lai et al. 2023; Guo et al. 2024). To our knowledge, there are currently no reviews broadly summarizing the reported PLA-degrading enzymes, despite their high potential for waste disposal and upcycling of widely used PLA. Here, we comprehensively review numerous purified PLA-degrading enzymes and summarize their characteristics in detail, including relevant methods that can be used to study the degradation process. Furthermore, we emphasize the potential for enhancing reaction yields using machine learning techniques and protein engineering, with the goal of optimizing catalytic performance.

### Biodegradation of PLA

While bioplastics are considered eco-friendly, their use can still cause environmental effects. Therefore, waste disposal and recycling should be taken into consideration, especially given their increasing use as in the case of PLA. Although PLA is classified as a biodegradable polymer, it is mostly resistant to the attack of microorganisms (Teixeira et al. 2021). Its complete degradation in a natural environment may require several years, both on land and in the sea, and a few months under composting conditions (Rosli et al. 2021; Krasowska and Heimowska 2023). Moreover, PLA-degrading microorganisms are not very abundant in nature and may differ under different environmental conditions (Qi et al. 2018; Ncube et al. 2020). Therefore, there is a great interest in discovering and understanding degradation mechanisms. Studies have been conducted on four mechanisms for PLA degradation: chemical hydrolysis, thermal degradation, photodegradation, and microbial and enzymatic degradation (Zaaba and Jaafar 2020; Teixeira et al. 2021).

The degradation of PLA depends on its properties, such as enantiomeric composition, crystallinity, and molecular weight. Enantiomerically pure PLA (PLLA or PDLA) is relatively difficult to degrade in natural environments, due to the high crystallinity and, accordingly, high melting point (T_m_), and glass transition temperature (T_g_) (Urbanek et al. 2020). Highly crystalline PLA residues have shown prominent resistance toward enzymatic biodegradation which was inhibited above a critical level of crystallinity (Cai et al. 1996). Small changes in stereochemical composition may have a large effect on crystallinity and consequently may affect the degree of biodegradability (Reeve et al. 1994). Additionally, relatively low molecular weight polymers are usually more degradable compared to high molecular weight polymers, due to higher accessibility of the chain end groups (Reeve et al. 1994; Tokiwa and Calabia 2006). Higher surface area may also enhance degradation rates. Reducing PLA powder particles size has been shown to result in improved enzymatic hydrolysis (Hino et al. 2023). When it comes to microbial and enzymatic biodegradation, different PLA-degrading enzymes exhibit selectivity toward various PLA stereochemical forms based on their natural substrate selectivity (Reeve et al. 1994; Urbanek et al. 2020). Furthermore, in some cases, the repeat unit sequence distribution can affect enzymatic degradation rates as well. PLA films synthesized by copolymerizations of (l,l)-lactide and (d,l)-lactide underwent lower enzymatic degradation compared to PLA films synthesized by copolymerizations of (l,l)-lactide and (d,d)-lactide, with the same L monomer content (MacDonald et al. 1996). Recently, a rapid biodegradation of high molecular weight and crystalline PDLLA films was achieved using a defined mixture of microorganisms, producing different types of PLA depolymerases (Mistry et al. 2022).

In addition to the polymer properties, the conditions of the reaction have a large effect on degradation rates, with temperature being one of the main key parameters (Itävaara et al. 2002; Mu et al. 2021; James-Pearson et al. 2023). Glass transition temperature (T_g_) for semi-crystalline or amorphous PLA can range from 50 to 70 °C (Taib et al. 2023). High temperatures near the T_g_ can make the polymer chains more flexible and accessible to water and enzymatic attack, thus increasing the degradation rate (Karamanlioglu and Robson 2013; James-Pearson et al. 2023). Thermophilic conditions above 50 °C improve PLA biodegradation rates compared to lower temperatures of 37 °C and below (Itävaara et al. 2002; Karamanlioglu and Robson 2013). Additionally, thermal pretreatment of PLA could significantly enhance biodegradation (Mu et al. 2021).

As PLA degrades, it produces lactic acid which lowers pH over time. The resulting acidic conditions can limit biodegradation processes due to the denaturation of degrading enzymes (Cui et al. 2022a). To overcome the pH decline and enhance biodegradation rates, pH control, and adjustment must be considered. Dialysis or dilution were strategies shown to address this crucial issue (Nakamura et al. 2001; Panyachanakul et al. 2019; Hegyesi et al. 2019; Cui et al. 2022a). Recently, a continuous buffering method for enzymatic degradation of PLA, scalable to large volumes, was developed (Polyák et al. 2023). Through parallel solubility and acid-based equilibria of calcium carbonate in the degradation medium, the pH level remained constant, allowing continuous degradation.

### Microbial degradation

Microorganisms that were found to degrade PLA usually belong to three main domains: actinomycete, bacteria, and fungi. Due to the large size and low solubility of PLA chains, the degradation begins with the secretion of extracellular depolymerases that generate lactic acid products (monomers or oligomers). The soluble products can be subsequently transported inside the cells and utilized. The addition of substances such as gelatin, elastin, silk fibroin, or peptides, which are usually natural substrates for PLA-degrading enzymes, can induce their production and increase microbial degradation rates (Zaaba and Jaafar 2020). PLA-degrading microorganisms are reviewed in more detail by Xu et al. (2022a).

### Enzymatic degradation

Over the years, several PLA-degrading enzymes, mostly α/β hydrolases, have been isolated from various microorganisms, including proteases (EC 3.4), lipases (EC 3.1.1.3), cutinases (EC 3.1.1.74), and esterases (EC 3.1.1.1) (Qi et al. 2017; Xu et al. 2022a). Most of them have been purified from the culture supernatant of the original microorganisms, while several have also been recombinantly expressed in *Escherichia coli* or other bacterial and yeast expression systems. Several were discovered as PLA-degrading based on their degradation activity toward other polyesters. Like other polyester depolymerases, the degradation activity by PLA-degrading enzymes requires their ability to accommodate a large and highly hydrophobic substrate and hydrolyze the ester bond. A summary of the reported PLA-degrading enzymes is presented in Table 1, including parameters describing reaction conditions, type of PLA used as substrate, and methods used for the detection of the degradation products.

**Table 1:** PLA-degrading enzymes and properties of the respective reactions

Enzyme	Microbial origin	PLA substrate	Reaction conditions	Evaluation methods for PLA degradation	Degradation products	Remarks	References
Proteases							
Proteinase K	*Tritirachium album*	PLLA powder	Tris–HCL buffer (pH 8.0), 37 °C	• Lactic acid assay with p-hydroxydipheny • TLC • pH measurement • Weight loss	l-Lactic acid monomers and oligomers	• Can be commercially purchased • PDB: 1IC6	(Williams 1981)
		PDLLA (96% L, Mn 137 kDa) films	Tris–HCL buffer (pH 8.6), 37 °C	• DSC • WAXS • SEM • Weight loss	PLA residues		(Cai et al. 1996)
		PDLLA films (different L contents, Mw 40–162 kDa)	Tris–HCL buffer (pH 8.6), 37 °C	• SEM • Weight loss • Size exclusion chromatography	PLA residues		(Li et al. 2000b)
		PLA films (Mw 233 kDa)	-	• HPLC	Lactic acid monomers		(Oda et al. 2000)
		PLLA films (Mn 410 kDa)	Tris–HCL buffer (pH 8.5)	• Quartz crystal microbalance (QCM) • AFM	PLA residues		(Yamashita et al. 2005)
		PDLLA emulsion (98% L, Mw 156 kDa)	Phosphate buffer (pH 7.5). 27 °C	• GPC	Lactic acid monomers and PLA residues		(Watanabe et al. 2007)
		PLLA films (Mn 125 kDa, Mw 227 kDa)	Tris–HCL buffer (pH 8.6), 37 °C	• Weight loss • WAXS • SEM • GPC	PLA residues		(Huang et al. 2020)
		PDLLA films (different L/D ratios, Mn 44.6–85.7 kDa, Mw 86–167.1 kDa)	Tris–HCL buffer (pH 8.6), 23–60 °C	• Weight loss • GPC	PLA residues		(Cui et al. 2022a)
PLD	*Amycolatopsis* sp. strain K104-1	PLA emulsion (Mn 220 kDa)	Phosphate buffer (pH 7.1), 37 °C	• Turbidity decreasing • TLC	Lactic acid monomers and PLA residues	• Cloned and expressed in *Streptomyces lividans* 1326	(Nakamura et al. 2001)
		PLA film (Mn 220 kDa)	Phosphate buffer (pH 7.0), 37°C	• Weight loss • SEM	PLA residues		
		PLA emulsion (Mn 220 kDa)	Tris–HCl buffer (pH 8.0), 37 °C	• Turbidity decreasing • TLC • H-NMR • l-Lactic acid dehydrogenase assay • HPLC • MS	l-Lactic acid oligomers, dimers, and monomers, and PLA residues		(Matsuda et al. 2005)
PLAse I PLAse II PLAse III	*Amycolatopsisorientalis* ssp.* orientalis*	PLLA emulsion (Mw 85–160 kDa)	Phosphate buffer (pH 7.0), 50 °C	• SBA-40C lactate biosensor	l-Lactic acid monomers	• Higher efficiency than proteinase K	(Li et al. 2008)
		PLLA emulsion (Mn 40 kDa)	Phosphate buffer (pH 7.0), 50 °C	• SBA-40C lactate biosensor	l-Lactic acid monomers		(Li et al. 2013)
		PLLA granules (Mn 40 kDa)	Phosphate buffer (pH 8.0), 45 °C	• SBA-40C lactate biosensor • Weight loss	l-Lactic acid monomers and PLA residues		
Protease from T16-1	*Actinomadura keratinilytica* T16-1	PLLA emulsion	Tris–HCl buffer (pH 9.0), 60 °C	• Turbidity decreasing	PLA residues	• Biodegradation process was scaled up in a 5 L bioreactor	(Sukkhum et al. 2009b, 2009a)
		PDLLA emulsion (80% L, Mw 43kDa)	Tris–HCl buffer (pH 9.0), 60°C	• Turbidity decreasing • HPLC	Lactic acid monomers and PLA residues		(Youngpreda et al. 2017)
		PDLLA powder (80% L, Mw 43 kDa) and commercial PLA trays (90% PLA)	Tris–HCl buffer (pH 9.0), 60 °C	• HPLC • Weight loss	Lactic acid monomers and PLA residues		(Panyachanakul et al. 2019)
Protease from LP175	*Laceyella sacchari* LP175	PLLA emulsion	Tris–HCl buffer (pH 9.0), 60 °C	• Turbidity decreasing	PLA residues		(Hanphakphoom et al. 2014)
*Bp*AprE	*Bacillus pumilus*	PLLA films (Mw 186 kDa)	Tris buffer (pH 8.0), 30 °C	• Enzymatic l-lactate assay	l-Lactic acid monomers	• Cloned and expressed in *B. subtilis* • Site-directed mutagenesis identified essential residues and yielded more efficient mutants	(Cannon and Reynolds 2023)
Cutinases/lipases							
CLE (cutinase-like enzyme)	*Cryptococcus* sp. S-2	PLA emulsion (Mw 140 kDa)	Tris–HCl buffer (pH 8.0), 30 °C	• Turbidity decreasing	PLA residues	• PDLA preference • Cloned and expressed in *S. cerevisiae* • PDB: 2CZQ • Higher efficiency than proteinase K	(Masaki et al. 2005)
		PLLA and PDLA emulsions	Phosphate buffer (pH 7.0), 30 °C	• Turbidity decreasing • GPC • MS	Linear or cyclic lactic acid oligomers and PLA residues		(Kawai et al. 2011)
		PDLA and PDLLA films	Phosphate buffer (pH 7.0), 37 °C	• GPC • DSC • WAXS	PLA residues		
		PLLA and PDLA emulsions	Phosphate buffer (pH 7.0), 37 °C	• Turbidity decreasing	PLA residues		(Myburgh et al. 2023)
		PLLA and PDLA powders	Phosphate buffer (pH 7.0), 37 °C	• HPLC	Lactic acid monomers		
		PLLA and PDLA films	Phosphate buffer (pH 7.0), 37 °C	• HPLC • Weight loss • DSC • SEM	Lactic acid monomers and PLA residues		
PaE	*Pseudozyma antarctica* JCM 10317	PLLA films (Mw 130 kDa) and PDLLA films (Mw 20 kDa)	Tris–HCL buffer (pH 8.8), 30 °C	• SEM • TOC analyzer • d, l-lactic acid assay kit	d- and l-lactic acid monomers and PLA residues	• Cloned and expressed in *S. cerevisiae*	(Shinozaki et al. 2013b)
		PLLA films (Mw 186 kDa, Mn 113 kDa) and PDLLA films (Mw 11 kDa, Mn 4 kDa)	Tris–HCL buffer (pH 8.5), 25 °C	• SPR • AFM	PLA residues		(Shinozaki et al. 2013a)
CfCLE	*Cryptococcus flavus* GB-1	PLLA films (Mw 100kDa) and PDLLA films (Mw 20 kDa)	HEPES–NaOH buffer (pH 7.8), 30 °C	• TOC analyzer	Soluble lactic acid products	• PDLLA preference	(Watanabe et al. 2015)
Thc_Cut1 Thc_Cut2	*Thermobifida cellulosilytica*	PLLA films	Phosphate buffer (pH 7.0), 37 °C	• HPLC	Soluble lactic acid products	• Cloned and expressed in *E. coli* BL21 • PDB: 5LUI, 5LUJ • Site-directed mutagenesis identified essential residues and yielded more efficient mutants: Thc_Cut2_DM (PDB: 5LUK) and Thc_Cut2_TM (PDB: 5LUL)	(Ribitsch et al. 2017)
PlaA	*Paenibacillus amylolyticus* Strain TB-13	PDLLA emulsion (Mw 5–20 kDa)	Phosphate-NaOH buffer (pH 10.0), 37 °C	• Turbidity decreasing • TOC analyzer • d, l-lactic acid assay kit	d- and l-lactic acid monomers and oligomers and PLA residues	• Cloned and expressed in *E. coli* BL21 • Higher efficiency than proteinase K	(Akutsu-Shigeno et al. 2003)
Lipase from DS04-T	*Pseudomonas* sp. strain DS04-T	PLA emulsion (Mn 400 kDa)	Phosphate buffer (pH 8.0), 50 °C	• l-Lactic acid assay kit • GC–MS	l-Lactic acid monomers		(Wang et al. 2011a)
Lipase from MTCC 2594	*Aspergillus niger* MTCC 2594	PLA emulsion (Mw 5–20 kDa)	Phosphate buffer (pH 7.0), 30 °C	• Turbidity decreasing	PLA residues	• Cloned and expressed in *Pichia pastoris*	(Nakajima-Kambe et al. 2012)
LIP1 LIP2 LIP3 LIP4	*Pseudomonas chloroaphis* PA23	PLA emulsion	Not mentioned	• GPC	PLA residues	• Cloned and expressed in *E. coli* BL21	(Mohanan et al. 2022, 2023)
**Esterases**							
PlaM4 PlaM7 PlaM9	Metagenomic library	PDLLA emulsion (Mw 5/20 kDa) and PLLA (Mw 130kDa)	Phosphate buffer (pH 7.0), 30 °C	• Turbidity decreasing	PLA residues	• Cloned and expressed in *E. coli* BL21	(Mayumi et al. 2008)
PlaM4		PDLLA powder (Mw 5/20 kDa)	Phosphate buffer (pH 7.0), 30 °C	• TOC analyzer	Soluble lactic acid products		
ABO2449	*Alcanivorax borkumensis*	PDLLA emulsions (Mw 5–70 kDa) and Inego™ PLA emulsions (6400 and 4032)	Tris–HCl buffer (pH 8.0), 30 °C	• Agarose gel plate assay	PLA residues	• Cloned and expressed in *E. coli* BL21 • Site-directed mutagenesis identified essential residues	(Hajighasemi et al. 2016)
		PDLLA emulsion (Mw 10 kDa)	Tris–HCl buffer (pH 8.0), 30 °C	• Turbidity decreasing	PLA residues		
		PDLLA (Mw 10/18 kDa) powder	Tris–HCl buffer (pH 8.0), 35 °C	• Lactate dehydrogenase assay • LC–MS • GPC	Lactic acid monomers and oligomers, PLA residues		
RPA1511	*Rhodopseudomonas palustris*	PDLLA emulsions (Mw 5–70 kDa)	Tris–HCl buffer (pH 8.0), 30 °C	• Agarose gel plate assay	PLA residues	• Cloned and expressed in *E. coli* BL21 • PDB: 4PSU • Site-directed mutagenesis identified essential residues	(Hajighasemi et al. 2016)
		PDLLA emulsion (Mw 10 kDa)	Tris–HCl buffer (pH 8.0), 30 °C	• Turbidity decreasing	PLA residues		
		PDLLA powder (Mw 10/18 kDa)	Tris–HCl buffer (pH 8.0), 35 °C	• Lactate dehydrogenase assay • LC–MS • GPC	Lactic acid monomers and oligomers, PLA residues		
MGS0156	Metagenomic library	PDLLA emulsion (Mw 2–70 kDa)	Tris–HCl buffer (pH 8.0), 30 °C	• Agarose gel plate assay • Lactate dehydrogenase assay • LC–MS	Lactic acid monomers and oligomers, PLA residues	• Cloned and expressed in *E. coli* BL21 • PDB: 5D8M • Site-directed mutagenesis identified essential residues and yielded more efficient mutant	(Popovic et al. 2017; Hajighasemi et al. 2018)
GEN0105	Metagenomic library	PDLLA emulsion (Mw 2–70 kDa) PLLA emulsion (Mw 40 kDa Ingeo PLA6400 emulsion	Tris–HCl buffer (pH 8.0), 30 °C	• Agarose gel plate assay • Lactate dehydrogenase assay • LC–MS	Lactic acid monomers and oligomers, PLA residues	• Cloned and expressed in *E. coli* BL21	(Popovic et al. 2017; Hajighasemi et al. 2018)
Esterase from TKU015	*Pseudomonas tamsuii* TKU015	Inego PLA emulsion (Mn 12.5 kDa)	Phosphate buffer (pH 7.0), 50 °C	• l-Lactic acid assay kit	Lactic acid monomers		(Liang et al. 2016)
Esterase from strain S3	*Pseudomonas aeruginosa* S3	PLA film (Mw 200 kDa)	Tris–HCl buffer (pH 8.0), 37 °C	• LC–MS	Linear and cyclic lactic acid oligomers		(Noor et al. 2020)
Others							
PME	*Pseudomonas* sp. strain DS04-T	PLLA emulsion (Mn 40 kDa)	Phosphate buffer (pH 8.0), 50 °C	• l-Lactic acid assay kit	l-Lactic acid monomers	• Cloned and expressed in *E. coli* BL21	(Wang et al. 2011b)
		PLLA film (Mn 40 kDa)	Phosphate buffer (pH 8.5), 37°C	• Weight loss • SEM • GC/MS	l-Lactic acid monomers and PLA residues		

### Proteases

Although PLA contains ester bonds, it can be cleaved by proteases that hydrolyze peptide bonds. PLA-degrading proteases are mainly serine proteases due to the similarity between the structures of their native substrate fibroin and PLA. Fibroin is rich in l-alanine which has an analogous structure to l-lactic acid. Due to the l-stereochemistry of amino acids, serine proteases can degrade PLLA and PDLLA but cannot degrade PDLA (Tokiwa and Calabia 2006). Additionally, several alkaline proteases showed degrading activity toward PLA, while neutral proteases showed little activity and acid proteases showed no activity at all (Oda et al. 2000). Different commercial α-chymotrypsins from bovine pancreas were also found to degrade PLLA, implying their additional importance in biomedical applications of PLLA (Lim et al. 2005).

Proteinase K from the fungus *Tritirachium album* was first reported to degrade fibrous particles of PLLA in 1981 (Williams 1981). Since then, it is the most investigated PLA depolymerase and used as a gold standard for PLA enzymatic degradation. It has also been used extensively for the enzymatic degradation of PLA-based materials containing additives or PLA blends (Shi et al. 2019; Donate et al. 2020; Seok et al. 2022; Urinov et al. 2022; Cui et al. 2022b). Based on its crystal structure and sequence, the enzyme is a member of the subtilisin family (Betzel et al. 1988). It was found to degrade semi-crystalline PLLA films within a few days and amorphous PLLA films within a shorter time of a few hours since it hydrolyzes predominantly amorphous regions (Reeve et al. 1994; Yamashita et al. 2005; Xu et al. 2022b). Usually, PLA-degrading assays with proteinase K are performed at optimal conditions with alkaline pH of 8.6 and 37 °C (Reeve et al. 1994; MacDonald et al. 1996; Li et al. 2000b, 2000a). Recently, it has been reported that increasing temperatures up to 50 °C retained the depolymerizing activity, compared to 37 °C (Cui et al. 2022a). Moreover, proteinase K has been shown to have high thermal stability. When being in the dry bulk state, incubation of PLLA films with proteinase K at up to 130 °C resulted in non-significant activity loss (Xu et al. 2022b). Withstanding prolonged incubation at elevated temperatures can be used for self-degrading PLA films. Such films that can be degraded under natural conditions were developed by embedding proteinase K during processing with chloroform at 200 °C (Huang et al. 2020). The PLLA films, with embedded proteinase K, were fully degraded in buffer, while embedding proteinase K encapsulated in polyacrylamide resulted in faster degradation. In another study, embedding lyophilized proteinase K into PLA films, together with random heteropolymers, has also shown to facilitate their near complete degradation after one week (DelRe et al. 2021).

An additional extracellular protease, named PLD, was purified from the bacteria *Amycolatopsis* sp. strain K104-1 that was found to degrade PLA after screening soil samples (Nakamura et al. 2001). The enzyme was further cloned and expressed in *Streptomyces lividans* 1326 (Matsuda et al. 2005). Sequence analysis showed its belonging to the chymotrypsin family of serine proteases and the essential site for PLA degradation consists of His74, Asp111, and Ser197. The purified protease had a molecular weight of approximately 24 kDa and proteolytic activity was characterized using *p*-nitroanilides (Suc-(Ala)_n_-pNA) as substrates. It could degrade emulsified high molecular weight PLA into lactic acid oligomers and dimers, and eventually into monomers after up to 6 h at 37 °C. Degradation assays with high molecular weight PLA films resulted in more than 90% weight loss after 48 h of incubation. The enzyme could also degrade proteins such as casein and fibrin but not polyesters such as PHB and PCL. Additionally, it was quite stable in a wide range of pH values and degradation activity increased above pH 5.0 with maximal activity at pH 9.5. Thermostability decreased with incubation at increasing temperatures above the optimum of 55–60 °C.

Three different extracellular serine-like proteases were purified simultaneously from the PLA-degrading bacteria *Amycolatopsis orientalis* ssp. *orientalis* (Li et al. 2008)*.* The enzymes, named PLAase I, PLAase II and PLAase III, had molecular weights of 24.0, 19.5, and 18.0 kDa, respectively. All three enzymes showed significantly higher degrading activity against emulsified high molecular PLA, compared to the standard PLA-degrading enzyme proteinase K. Like PLD from strain K104-1, PLAses showed hydrolytic activity toward casein but not toward PHB. Additionally, similar to other PLA-degrading proteases, the optimal activity was achieved at alkaline pH of 9.5–10.5 and no activity was observed under acidic conditions. PLAase I and PLAase III showed high thermostability after incubation at temperatures below 60 °C for 8 h and their optimal activity was observed at this temperature. PLAase II had lower thermostability, being stable up to 50 °C, which was the optimal temperature. The fermentation parameters for the production of the crude enzymes by *A. orientalis* ssp. *orientalis* were optimized (Li et al. 2013). The main factors were found to be the time course of the fermentation and the concentrations of PLA and gelatin that served as carbon and nitrogen sources, respectively, as well as inducers. Incubation of PLLA granules with the crude enzymes, without a purification step, resulted in lactic acid release accompanied by more than 50% weight loss after 24 h, and complete degradation after 5 days.

Serine protease purified from the culture supernatant of thermophilic *Actinomadura keratinilytica* T16-1 exhibited high degrading activity toward emulsified pure PLA (the composition of which was not reported) (Sukkhum et al. 2009b). High activity was also observed toward *p*-nitroanilide (Suc-(Ala)_3_-pNA) and gelatin, whereas on casein showed comparatively lower activity, and there was no detectable activity toward PCL. Compared to other reported PLA-degrading enzymes, the purified enzyme was highly stable and active under alkaline conditions and high temperature with optimum at pH 10.0 and 70 °C, for 30 min of reaction. The production of the enzyme by *Actinomadura* sp. T16-1 was optimized in a 3-L airlift fermenter (Sukkhum et al. 2009a). Similar to PLAase production, the addition of PLA and gelatin as inducers and carbon and nitrogen sources was proven to be essential. The degradation process by the purified enzyme was also optimized for temperature, time of reaction and enzyme activity (Youngpreda et al. 2017). The degradation efficiency was similar to that of commercial proteinase K. The highest lactic acid concentration was obtained from PDLLA powder, with 82% degradation, following 24 h of incubation using the conditions of 200 U/ml of activity at 60 °C. Another study focused on scale up of the biodegradation process by the enzyme in a 5-L bioreactor (Panyachanakul et al. 2019). Slow agitation of 50 rpm at 60 °C and pH 8.0 resulted in 89% conversion efficiency after 72 h. Simultaneous PLA degradation and dialysis improved the process by removing lactic acid to prevent pH decline. This method resulted in almost 100% conversion efficiency of PLA powder and 32% weight loss of commercial PLA tray.

Another serine protease, isolated from the thermophilic filamentous bacteria *Laceyella sacchari* LP175, was also found to degrade emulsified PLLA with high molecular weight (Hanphakphoom et al. 2014). In addition to PLLA, it showed strong activity against casein and gelatin but weak activity against *p*-nitroanilide. The purified enzyme, with molecular weight of approximately 28 kDa, was stable at temperatures up to 50 °C and in pH values ranging from 8.5 to 10.5. The optimum activity was obtained with pH of 9.0 and 60 °C, near the T_g_ of PLA. Additionally, it was used for the degradation of PLA/thermoplastic starch (PLA/TPS) films as a mixture with raw starch-degrading enzyme (Lomthong et al. 2022).

Recently, a subtilisin from *Bacillus pumilus*, named *Bp*AprE, was studied for its ability to degrade high molecular PLLA films (Cannon and Reynolds 2023). The enzyme was recombinantly expressed in* Bacillus subtilis* with a molecular weight of 27.8 kDa. Reaction was performed at 30 °C under alkaline conditions of pH 8.0 and l-lactate was released and measured for 15–17 h. To identify the essential residues for PLA degradation, a comparative analysis with non-PLA-degrading homologous subtilisin from *B. subtilis* (*Bs*AprE) was performed. Based on sequence alignment and the crystal structure of *Bs*AprE, site-directed mutagenesis was performed to make it more similar to active *Bp*AprE. Along with computational models, the importance of hydrophobic surface and large binding pocket was demonstrated, and several variants were obtained for both subtilisins (Ser33Thr/Thr99Tyr/Glu156Ser for *Bs*AprE and Asp101Ser/Asn130Thr/Ser189Phe for* Bp*AprE), exhibiting significantly higher activities compared to the wild types.

### Lipases and cutinases

Lipases can catalyze PLA ester bond hydrolysis. Cutinases, mostly produced by fungi, hydrolyze the polyester cutin on the surface of plants. Studies have shown that cutinase-like enzymes can additionally degrade PLA (Urbanek et al. 2020). Compared to proteases, lipases and cutinases are not enantioselective, most likely because their substrates have no chirality. However, they usually show preference toward the PDLA stereochemical form and racemic PDLLA (Kawai et al. 2011).

A cutinase-like enzyme (CLE) isolated from the yeast *Cryptococcus* sp. S-2 showed degrading activity toward high molecular PLA, as well as other bioplastics including PCL and PBS (Masaki et al. 2005). It could degrade both PDLA and PLLA emulsions and films, with preference toward PDLA. Emulsified PDLA was degraded almost completely after approximately 24 h of incubation with the purified enzyme at 30 °C (Kawai et al. 2011). Interestingly, it showed significant higher efficiency for PLA degradation compared to proteinase K, with higher V_max_ toward PLLA (Masaki et al. 2005; Kawai et al. 2011). The crystal structure of the enzyme was determined and showed α/β hydrolase fold with catalytic triad of Ser85, Asp165, and His180, as well as stabilizing disulfide bonds. Site-directed mutagenesis was performed to enhance its hydrolytic activity toward long-chain substrates by increasing the size of the hydrophobic residues (Kodama et al. 2009). Recently, the enzyme was recombinantly expressed in *Saccharomyces cerevisiae* after codon optimization (Myburgh et al. 2023). Similar to the purified enzyme from the wild-type host, the crude supernatant containing the secreted recombinant enzyme efficiently degraded both PDLA and PLLA emulsions, powders and films, with PDLA preference. Near complete hydrolysis of both PDLA and PLLA emulsions was achieved after 72 h, while approximately 40% weight loss was achieved for PLLA and PDLA films after 10 days.

Another CLE, named PaE, was purified from *Pseudozyma antarctica* JCM10317 which was previously found to degrade PBS and poly(butylene succinate-co-adipate) (PBSA) (Shinozaki et al. 2013b). The encoding gene was isolated, and the enzyme was expressed in *S. cerevisiae*. In addition to PLLA and PDLLA films, the recombinant enzyme could degrade as well as films of PBS, also PBSA and PCL. Characterization of the enzyme activity with emulsified PBSA as substrate showed optimal activity at pH 9.5 and 40 °C, while stability sharply decreased after incubation at this temperature for 1 h. After incubation with PaE at 30 °C for 24 h, the degradation rate of the PLA films was 50.4%. The comparison of d- and l-lactic acid monomers content and total organic carbon released indicated a random endo-type cleavage mechanism of PaE. Additionally, surface plasmon resonance analysis showed strong adsorption of PaE onto the surface of amorphous PLLA films (Shinozaki et al. 2013a).

A CLE isolated from *Cryptococcus flavus* GB-1 was reported to degrade emulsified PBSA (Watanabe et al. 2015). The enzyme had a molecular weight of approximately 22 kDa and the amino acids sequence was found to be 93% identical to that of CLE from *Cryptococcus* sp. S-2; therefore, it was named CfCLE. Evaluation of the enzymatic degradation of PLA by CfCLE showed more efficient activity toward PDLA compared to PLLA film, similar to other CLEs. Degrading activity was also observed toward PCL and PBSA films. Biochemical characterization with emulsified PBSA showed the optimal activity at pH 7.8 and 45 °C, while the stability of the enzyme sharply decreased above this temperature.

In addition to fungus CLEs, bacterial cutinases from *Thermobifida cellulosilytica* also exhibited the ability to degrade PLA. Recombinant Thc_Cut1 and Thc_Cut2, expressed in *E. coli*, were previously found to hydrolyze PET. The two enzymes had molecular weights of 29.4 and 29.7 kDa, respectively, and showed high homology (Herrero Acero et al. 2011). In a subsequent study, the two wild-type cutinases and two variants of Thc_Cut2 (Thc_Cut2_DM: Arg29Asn/Ala30Val and Thc_Cut2_TM: Arg19Ser/Arg29Asn/Ala30Val) have also been shown to degrade PLLA films at 37 °C and neutral pH of 7.0 (Ribitsch et al. 2017). Thc_Cut2 variants were obtained by the replacement of amino acids, based on the differences in the electrostatic and hydrophobic surface properties compared to Thc_Cut1, which previously showed higher efficiency of PET hydrolysis (Acero et al. 2013). The two variants exhibited higher activity toward PLLA films, releasing higher amounts of lactic acid compared to the wild type. The crystal structures of the wild-type cutinases and the variants were determined, and their mechanisms were analyzed. All of them had α/β hydrolase fold with a catalytic triad of Ser131, Asp177, and His 209. On the other hand, the surface regions were further investigated, shedding more light on the essential uncharged and hydrophobic amino acids for PLA degradation.

A gene encoding a PLA-degrading lipase, named PlaA, was isolated from a genomic library of *Paenibacillus amylolyticus* strain TB-13 and recombinantly expressed in *E. coli* BL21 (Akutsu-Shigeno et al. 2003). The purified enzyme had a molecular weight of approximately 22 kDa and it could degrade emulsified PDLLA, as well as other polyesters such as PBS, PBSA, and PCL. Degrading activity was higher toward lower molecular weight PLA (below 10,000 g/mol), achieving complete degradation after 30-min incubation at 37 °C, compared to 90 min for the higher molecular weight PLAs. Notably, PlaA was more active than proteinase K, with higher and faster degradation rates. Moreover, PlaA also exhibited lipase activity toward triolein and esterase activity toward tributyrin and *p*-nitrophenyl alkyl esters. The enzyme stability and PLA-degrading activity were higher at alkaline pH values with optimum at pH 10.0. The maximal activity was observed at 45–55 °C, while stability decreased above 50 °C.

In another study, lipase was purified from the cell free supernatant of *Aspergillus niger* MTCC 2594 and recombinantly expressed in *Pichia pastoris* (Nakajima-Kambe et al. 2012). Both native and recombinant enzymes showed high degrading activity toward low molecular emulsified PLA, with up to 87% degradation within less than 24 h at 30 °C and pH 7.0. Furthermore, degrading activity was also exhibited toward PCL. Based on SDS-PAGE analysis, the recombinant enzyme had molecular mass of 35 and 37 kDa, higher than the predicted, indicating post-translational modifications. Characterization of lipase activity using olive oil as a substrate revealed optimal conditions at 37 °C and pH 7.0. Moreover, the enzyme exhibited stability across a broad pH range, from 4.0 to 10.0, and remained functional up to 50 °C.

A lipase purified from the culture supernatant of *Pseudomonas* sp. strain DS04-T was found to degrade emulsified PLLA into lactic acid monomers (Wang et al. 2011a). Additionally, it exhibited degradation activity toward PCL and PHB. The purified enzyme had a molecular weight of 34.0 kDa. It was quite stable in a wide pH range of 3.5–10.0 and up to 60 °C, when optimal depolymerizing activity was achieved at 50 °C and pH 8.5. Another PLA depolymerase from *Pseudomonas* sp. strain DS04-T, named PME, was identified by screening genomic libraries for growth on emulsified PLLA (Wang et al. 2011b). PME showed degrading activity against PLLA films, but did not exhibit lipase, esterase, protease, or PCL and PHB depolymerase activity. Sequence analysis revealed homology with Nuclear Transport Factor 2 (NTF2-like) family of proteins that share structural similarities but have diverse functions. The encoding gene was cloned and expressed in *E. coli* BL21, exhibiting a molecular weight of 19.2 kDa. Similar to the previously mentioned PLA depolymerase from *Pseudomonas* sp. strain DS04-T, the purified recombinant PME was stable across a wide pH range. However, activity was retained up to higher temperatures of 80 °C. Optimal depolymerizing activity was also observed at higher temperatures of 60 °C and pH 8.5.

Genes encoding four PLA-degrading lipases, named LIP1-LIP4, were recently isolated from *Pseudomonas chlororaphis* PA23 that was previously found to hydrolyze PHA polymers (Mohanan et al. 2022, 2023). Interestingly, unlike most PLA-degrading enzymes, LIP1-LIP3 are intracellular enzymes, while LIP4 is an extracellular enzyme. The enzymes were recombinantly expressed *E. coli* showing molecular weights of approximately 30 kDa. Then, they were evaluated for their degradation activity toward several biodegradable polymers, including PLA. Incubation with the lipases for 96 h resulted in the decrease of the molecular weight of PLA powder suspension. Characterization of the activities with *p*-nitrophenyl octanoate (PNPO) as substrate showed optimal activity at pH 6.0 for LIP4, compared to alkaline pH of 8.0 or 9.0 for the LIP1-3. The optimal temperatures were 45 °C for both LIP1 and LIP3, 40 °C for LIP2 and 50 °C for LIP4. All four lipases were thermostable retaining at least 70% of their activities after incubation at 45 °C for 24 h.

### Esterases

Esterases are enzymes that can break down ester bonds using a diverse range of substrates. They typically belong to the α/β fold group, much like lipases. However, esterases are different from lipases in that they tend to exhibit higher activity toward more soluble substrates, while lipases exhibit higher activity toward hydrophobic substrates (Fojan 2000).

Many microbial strains cannot be cultured under laboratory conditions; therefore, there is difficulty in characterizing such microorganisms as PLA-degrading strains and isolating the degrading enzymes. Three different PLA-degrading enzymes, named PlaM4, PlaM7 and PlaM9, were identified based on the screening of a metagenomic library constructed of DNA extracted from PLA disks buried in compost (Mayumi et al. 2008). The enzymes were characterized following their cloning and expression in *E. coli*. Their molecular weights were approximately 48, 30, and 38 kDa, respectively. All of them could degrade emulsified low molecular PDLLA in 30 min at 30 °C, while PlaM4 showed the highest activity with more than 50% clearance. Based on their substrate specificity against tributyrin, the enzymes were characterized as carboxylesterases. They also showed degrading activity against other polyesters such as PBS, PBSA, polyethylene succinate (PES), and PCL, as well as short-chain fatty acids. Due to its higher activity and thermophilic properties that are most suitable for PLA degradation, PlaM4 was further investigated. Optimal activity was observed at a high temperature of 70 °C and pH 7.0, with high stability at up to 60 °C.

Esterases ABO2449 from *Alcanivorax borkumensis* and RPA1511 *Rhodopseudomonas palustris* were discovered as efficient depolymerases of emulsified and solid PLA following screening of 90 purified uncharacterized microbial α/β hydrolases (Hajighasemi et al. 2016). Additionally, they also exhibited hydrolytic activity against PCL and other polyesters. The enzymes were recombinantly expressed in *E. coli* and then characterized for their PLA-degrading activity. Both enzymes hydrolyzed only racemic PDLLA but not pure PDLA and PLLA with high crystallinity. ABO2449 exhibited greater hydrolytic activity compared to RPA1511, resulting in almost complete degradation of emulsified PLA within an hour, and solid PLA within 36 h. The addition of detergent facilitated its binding to the hydrophobic surface of solid PLA, resulting in significantly enhanced degradation rates. Esterase activity assays with α-naphthyl propionate showed optimal activity at pH 9.5–10.0 for both enzymes. Regarding temperature, the optimum was 30–37 °C for ABO2449 and 55–60 °C for RPA1511. ABO2449 showed lower thermostability when aggregated at 32.3 °C, compared to RPA1511 that had higher aggregation temperature of 70.8 °C. Based on the analysis of the degradation products, the enzymes could perform both exo- and endoesterase cleavage of PLA. The crystal structure of RPA1511 was determined, and together with site-directed mutagenesis, it revealed the serine hydrolase catalytic triad consisting of Ser114, Asp242 and His270. In addition, the essential amino acids required for hydrolyzing PLA substrates, but not soluble monoester substrates, were identified. They included both hydrophobic and polar or charged residues, bonded with methyl and carbonyl groups on the side chains of PLA.

Other PLA-degrading carboxylesterases were discovered by screening purified enzymes from broader metagenomic libraries, that were previously screened for tributyrin degradation (Popovic et al. 2017). Of these esterases, the most prominent PLA degrading were the serine α/β hydrolases GEN0105 and MGS0156, from uncultured organisms (Hajighasemi et al. 2018). Both enzymes could degrade emulsified PDLLA with a broad range of molecular weights, as well as polylactic-co-glycolic acid (PLGA), PCL, and PBSA. However, enantiomerically pure PDLA could not be degraded, while PLLA and commercial Ingeo PLA6400 were only degraded by GEN0105. Both enzymes showed mesophilic activity with maximum at 40 °C and 35 °C for MGS0156 (with α-naphthyl butyrate as substrate) and for GEN0105 (with α-naphthyl propionate as substrate), respectively. While both being active at alkaline pH, MGS0156 showed higher acidity tolerance. Monomeric and oligomeric lactic acid products revealed endo- and exoesterase activity of the enzymes. MGS0156 showed high degrading activity toward solid PDLLA powder with 50% conversion within 3 h and almost complete degradation after 18 h. The crystal structure of tetrameric MGS0156 was solved and the positions of the active sites, consisting of Ser232, His373, and Asp350 catalytic triad, were determined. Additionally, it showed the hydrophobic lid domain that is stabilized by a disulfide bond. A structural computational model of GEN0105 revealed its catalytic triad consisting of Ser168, His292 and Glu262. Site-directed mutagenesis of MGS0156 yielded a variant (Leu169Ala) with higher activity (90% conversion within only 3 h) compared to the wild type, achieved by reducing the size of the residue adjacent to catalytic site.

PLA depolymerase, which had an esterase activity and a molecular weight of approximately 58 kDa, was purified from the culture supernatant of *Pseudomonas tamsuii* TKU015 with (Liang et al. 2016). The enzyme could degrade emulsified PDLLA and showed hydrolytic activity against fibrinogen and tributyrin. It was quite stable across a wide pH range of 5.0–10.0 and up to 80 °C. The PLA-depolymerizing activity increased with increasing pH and optimal activity was observed at pH 10.0 and 60 °C, near the T_g_.

Another esterase was purified from the supernatant of the PLA-depolymerizing bacteria *Pseudomonas aeruginosa* strain S3, with a molecular weight of 34.0 kDa (Noor et al. 2020). The purified esterase could degrade PLA films at 37 °C, releasing linear and cyclic lactic acid oligomers after 72 h of reaction. Characterization of the enzyme showed high activity and stability at up to 50 °C and pH range of 5.0–8.0, with optimal conditions of 30 °C and pH 7.0. The activity was enhanced in the presence of non-ionic surfactants that increase the interfacial area with the hydrophobic PLA. Stability was observed in several organic solvents such as glycerol, propanol, and acetone, which can be beneficial for industrial applications.

### Analytical methods for measuring PLA degradation

PLA degradation is usually evaluated using emulsified polymer or solid polymer films as substrates. The degradation products are classified as either insoluble fractions consisting of remaining PLA residues or soluble products comprising lactic acid oligomers or monomers.

### Evaluation of insoluble products

When emulsified PLA is degraded into soluble lactic acid products, turbidity declines, and the degradation rates of PLA can be evaluated. Qualitative analysis can be performed using the agarose plate assay. A PLA emulsion is solidified by adding agarose to form a turbid gel inside cell culture dishes. A cell sample or mutant library can be seeded onto the plates. In the case of purified enzymes, small wells can be punched in the gel, which will be further loaded with a tested enzyme (Popovic et al. 2017; Hajighasemi et al. 2018). A translucent halo around the formed colonies or the wells containing degrading enzymes indicates the degradation of PLA into soluble products. This method can be used for high throughput screening to isolate PLA-degrading strains or PLA-degrading enzymes. A more quantitative method, which can also be used for high throughput screening, is measurement of turbidity of a liquid PLA emulsion and monitoring the decrease in optical density at typical wavelengths of 580–660 nm (Nakamura et al. 2001; Akutsu-Shigeno et al. 2003; Masaki et al. 2005; Sukkhum et al. 2009b; Sukkhum et al. 2009a; Kawai et al. 2011; Nakajima-Kambe et al. 2012; Hanphakphoom et al. 2014; Hajighasemi et al. 2016; Myburgh et al. 2023).

A direct method for the evaluation of degradation of PLA films is monitoring their weight loss following incubation with the tested enzyme and removal of the soluble fraction (Cai et al. 1996; Li et al. 2000b, 2013; Nakamura et al. 2001; Panyachanakul et al. 2019; Huang et al. 2020; Cui et al. 2022a). Yet, degradation products can still be found in the insoluble fraction when shorter polymer chains are formed. Gel permeation chromatography (GPC) is performed to analyze the obtained number average molecular weight (Mn), weight average molecular weight (Mw), and polydispersity (Mw/Mn), which indicates the homogeneity of the polymer chains molecular weights (Li et al. 2000b, 2000a; Kawai et al. 2011; Watanabe et al. 2015; Hajighasemi et al. 2016; Huang et al. 2020; Mohanan et al. 2022, 2023). Other methods can be used to evaluate the new mechanical and thermal properties of the degraded PLA. Scanning electron microscopy (SEM) is used for monitoring the morphology of PLA films’ surface. The initial surface is usually clear and smooth, while during degradation it is rougher and appears with holes and cracks that can lead to disintegration of the PLA films (Li et al. 2000a; Nakamura et al. 2001; Noor et al. 2020; Huang et al. 2020; Myburgh et al. 2023). Differential scanning calorimetry (DSC) analysis is performed to determine the melting point (T_m_) and glass T_g_ of PLA films, indicating the degree of crystallinity (Cai et al. 1996; Li et al. 2000b, 2000a; Kawai et al. 2011; Myburgh et al. 2023). Wide angle X-ray (WAXS) analysis is also used to determine the degree of crystallinity (Cai et al. 1996; Kawai et al. 2011; Huang et al. 2020). These methods are used for determining the influence of the initial degree of crystallinity on degradation rates. Additionally, changes in the degree of crystallinity can indicate the degradable areas of the PLA films. Since degradation of amorphous regions is usually preferable, crystallinity degree can increase during the beginning of the degradation reaction and then decrease due to slower and advanced degradation of crystalline regions (Kawai et al. 2011). Atomic force microscopy (AFM) is used to evaluate various mechanical and surface properties of polymer films. For evaluation of PLA degradation, it was used to analyze the degraded PLA surface morphology and erosion depth by the degrading enzyme (Shinozaki et al. 2013a). In another study, it was used for the determination of PLA thin-film thickness before and after incubation with a depolymerase (Yamashita et al. 2005).

### Evaluation of soluble products

Soluble lactic acid products usually consist of either monomers, which can be divided into l- and d- enantiomers, or lactic acid oligomers, which can differ in their length and conformation (linear or cyclic). Various methods have been developed over the years for the detection of lactic acid (Rattu et al. 2021). Chromatography methods, such as thin layer chromatography (TLC) (Williams 1981; Nakamura et al. 2001; Matsuda et al. 2005) and high-performance liquid chromatography (HPLC) (Oda et al. 2000; Matsuda et al. 2005; Ribitsch et al. 2017; Youngpreda et al. 2017; Panyachanakul et al. 2019; Myburgh et al. 2023), are used to separate and distinguish between lactic acid monomers and different oligomers. Employing a chiral HPLC column can distinguish between the two enantiomers. Further methods, such as mass spectrometry (MS) (Matsuda et al. 2005; Wang et al. 2011a; Kawai et al. 2011; Hajighasemi et al. 2016, 2018; Noor et al. 2020) and H-nuclear magnetic resonance (H-NMR) (Matsuda et al. 2005), are also performed to determine the specific composition. Lactic acid monomers can be also detected by enzymatic assays and kits based on lactate dehydrogenase reaction, and subsequent fluorometric measurement of NADH product or coupling with a chromogenic reaction (Hajighasemi et al. 2016, 2018; Cannon and Reynolds 2023). These assays are selective toward lactic acid stereoisomers due to enzyme selectivity. Thus, a combination of d- and l-lactate dehydrogenase is necessary for the detection of both stereoisomers (Akutsu-Shigeno et al. 2003). Measurement of the total organic carbon (TOC) content of the soluble fraction also indicates the concentration of soluble lactic acid products, although it cannot distinguish between monomers and soluble oligomers. A combination of specific methods for the determination of the product composition can give insights into the hydrolysis mechanism of the enzyme (endo- or exo- type cleavage) (Shinozaki et al. 2013b). Another indirect method for monitoring PLA degradation is by pH measurement or titration during the reaction (Williams 1981; Miksch et al. 2021). As the process progresses, the pH is expected to decline with the formation of lactic acid.

### Outlook for PLA enzymatic degradation

The discovery of diverse PLA-degrading enzymes opens the door to an efficient, low-cost, and sustainable approach to waste disposal and upcycling. Whole-cell fermentation approaches for PLA biodegradation, such as composting, mainly end with the assimilation of lactic acid products by microorganisms. In contrast, enzymatic degradation to the monomeric building blocks can be further employed to utilize the valuable feedstocks, thus maintaining a circular economy (Acosta and Alper 2023). In a circular economy, the materials re-enter the economy at the end of their use. By doing this, the linear take-make-waste system is transformed and made circular. Exploiting the liberated lactic acid can be routed for repolymerization into new PLA or other applications in different industries such as food, pharmaceutical, etc. While different PLA products contain one or several isomers of lactic acid, a mixture of enzymes with opposite specificity can be employed to fully depolymerize PLA into the monomers (Mistry et al. 2022).

Although having advantages over techniques such as composting, the use of enzymes for PLA degradation is still emerging and full degradation is difficult to reach, especially considering the chemically diverse and insoluble nature of the substrate. Further improvement through protein engineering can be performed to maximize enzymatic degradation performance and enhance activity and stability (Dror et al. 2014, 2015; Gihaz et al. 2020). Several strategies have been employed to optimize various plastic-degrading enzymes, mainly through rational design, based on known structures and computational approaches (Zhu et al. 2022; Acosta and Alper 2023). While mostly employed for polyethylene terephthalate (PET) hydrolases, these can also be implemented for bioplastics degrading enzymes such as PLA depolymerases. Analyses based on the structures and site-directed mutagenesis of PLA depolymerases emphasized the essential role of the wide binding site and hydrophobic surface interactions (Hajighasemi et al. 2016, 2018; Cannon and Reynolds 2023). A main factor affecting the efficiency of enzymatic degradation of polymers is the accessibility of the active site, which is affected by its width and flexibility (Fecker et al. 2018). Therefore, optimization of a wide and enlarged active site, that would effectively accommodate large substrates such as high molecular PLA, can improve enzymatic degradation. This strategy has been shown to increase the degradation activity of PLA-degrading subtilisin (Cannon and Reynolds 2023) and other polyester-degrading enzymes, such as cutinase and PET hydrolase (Bollinger et al. 2020; Dong et al. 2020). Along with the conformation of the active site, the surface interactions with the substrate are also critical. Higher hydrophobicity of surface residues, adjacent to the active site or in substrate binding sites, may contribute to higher affinity toward hydrophobic substrates such as PLA. Increasing surface hydrophobicity by replacement of charged amino acids with uncharged and hydrophobic amino acids in cutinase and PET hydrolase has been shown to result in higher degradation activity (Acero et al. 2013; Ma et al. 2018). Higher hydrophobic surface mutants of the cutinase Thc_Cut2 also showed high degrading activity against PLA (Ribitsch et al. 2017). In addition, since degradation is favorable at high temperatures near the T_g_ of PLA, in which polymer chains are more flexible, high thermostability of degrading enzymes is a crucial factor. Thermostability enhancement can be achieved through the addition of disulfide bridges (Gihaz et al. 2020), as was performed for PET degrading cutinase which showed an increase in its melting temperature (Tournier et al. 2020). Another route to improve PLA degradation activity and allow a continuous process is enhancing enzyme stability under acidic conditions that are formed due to the liberated lactic acid. Various techniques can be exploited for this purpose including surface charge and chemical modifications (Zhang et al. 2023). Exchange of uncharged residues on the surface of serine protease to negatively charged residues resulted in the shifting of optimal proteolytic activity toward lowered pH values (Jakob et al. 2013). In another study, serine protease activity and stability under acidic conditions were enhanced by conjugation to a polymer that can change its conformation depending on pH (Murata et al. 2013).

In addition to the improvement of known enzymes, new PLA-degrading enzymes, with desirable properties, can be discovered using novel bioinformatic computational prediction methods. As sequence similarity does not consistently correlate with the functional ability of enzymes to degrade plastics, a machine learning-based approach was recently developed for the prediction of such enzymes (Jiang et al. 2023). The system integrated data of a wide range of plastic-degrading enzymes, including PLA depolymerases like CLE and PlaM4, and could successfully establish sequence-based protein classification models.

## Conclusions

Considering the extensive use and wide-ranging applications of plastics in daily life and multiple industries, along with increased environmental concerns, PLA emerges as a promising biodegradable material and offers a potential substitute for polluting plastics. Nevertheless, the limited degradability of PLA in natural environments raises the need for faster and controlled degradation, to minimize the environmental impacts of its waste. Alongside traditional techniques such as mechanical recycling, composting, and the discovery of various PLA-degrading microorganisms, enzymes play a significant and promising role, especially in light of the desire to extend the life cycle of products. The review introduces diverse PLA-degrading enzymes that were isolated, purified, and characterized for their depolymerizing activity. Interestingly, different classes of hydrolases have been found to degrade PLA efficiently. However, high variability between the PLA samples and the respective evaluation methods results in a high discrepancy between the degradation rates and the hydrolytic outcome. The application of enzymes for PLA hydrolysis is still in the process of maturation toward full and upscaled degradation. Nonetheless, with improvements and stabilization through protein engineering methods, along with advances in molecular and fermentation methods, there is a great potential for utilizing such enzymes for more efficient PLA degradation processes. The use of enzymes can bypass the need for PLA waste purity in mechanical recycling processes due to their selectivity. Besides, enzymatic methods can result in lower environmental effects, consuming low energy. When using composting and other whole-cell approaches, PLA-degrading microorganisms assimilate degradation products for their own biomass growth or carbon dioxide production, thus hindering the reuse of PLA building blocks. Enzymatic degradation provides an efficient and sustainable technique for the upcycling and utilization of PLA building blocks for further purposes or additional polymerization and usage cycles. This approach is in line with the principles of a circular economy.

## Data Availability

Not applicable.
